# Determinants of oral health-related quality of life in orthodontic aligner wearers: A cross-sectional analysis

**DOI:** 10.1371/journal.pone.0319579

**Published:** 2025-03-13

**Authors:** Gabriela Luiza Nunes Souza, José Alcides Almeida de Arruda, Pâmella de Moura Dario, Esdras de Campos França, Marcelo de Araújo Lombardi, Giselle Cabral da Costa, Najara Barbosa Rocha, Lucas Guimarães Abreu

**Affiliations:** 1 Department of Child and Adolescent Oral Health, School of Dentistry, Universidade Federal de Minas Gerais, Belo Horizonte, Brazil; 2 Department of Oral Diagnosis and Pathology, School of Dentistry, Universidade Federal do Rio de Janeiro, Rio de Janeiro, Brazil; 3 Department of Orthodontics, Associação Brasileira de Odontologia, Belo Horizonte, Brazil; 4 Department of Social and Preventive Dentistry, School of Dentistry, Universidade Federal de Minas Gerais, Belo Horizonte, Brazil; University of Catanzaro, ITALY

## Abstract

Oral health-related quality of life (OHRQoL) is a critical measure for assessing the impact of orthodontic treatments, including aligners. This study aimed to investigate factors associated with OHRQoL among orthodontic aligner wearers. This cross-sectional study included 34 participants. OHRQoL was evaluated using the Oral Health Impact Profile (OHIP-14), and malocclusion severity was measure with the Dental Aesthetic Index (DAI). Data on sex, age, family income, and treatment duration were collected. Bivariate analyses were conducted to examine associations between independent variables and OHRQoL. Effect sizes (ES) were calculated to indicate the magnitude of associations, categorized as small, moderate, or large. Poisson regression was performed to identify factors associated with OHRQoL, reporting beta coefficients (Coef.) and standard errors (SE). Statistical significance was set at *p* < 0.05. The mean age of participants was 33.8 years. In the psychological discomfort subscale, males reported higher scores, with a moderate ES. Family income showed moderate-to-large ES, with individuals from families earning < 5 wages reporting higher scores. Higher DAI scores were significantly associated with poorer OHRQoL across the following subscales: functional limitation (*p* = 0.002), physical pain (*p* = 0.001), physical disability (*p* = 0.005), psychological disability (*p* = 0.003), social disability (*p* = 0.001), handicap (*p* = 0.037), and the total score (*p* = 0.001). Younger individuals also reported significantly higher scores on the handicap subscale (*p* = 0.007). Younger individuals and those with more severe malocclusion experienced poorer OHRQoL during treatment with orthodontic aligners. Sex and family income also significantly influenced OHRQoL outcomes.

## Introduction

Oral health-related quality of life (OHRQoL) refers to the extent to which oral health outcomes influence key aspects of daily living, including physical, emotional, and social well-being [1,2]. These effects can vary in duration, severity, and frequency, shaping individuals’ overall self-perception of their well-being [1,2]. While oral health conditions are a major determinant, OHRQoL is also influenced by a range of complex and multifactorial elements [3,4]. Age, sex, and socioeconomic status are consistently described in the literature as significant contributors [1–5]. For instance, older individuals often report poorer OHRQoL [5], whereas women generally report poorer quality of life (QoL) outcomes compared to men [6]. Moreover, socioeconomic disparities are well-documented, with individuals of lower socioeconomic status experiencing significantly worse OHRQoL [7]. These disparities emphasize the importance of addressing social determinants of health to reduce inequities in orthodontic care access and outcomes.

Orthodontic treatment, beyond improving dental function and esthetics, can profoundly impact overall health and provides psychosocial benefits [8,9]. Dental and skeletal discrepancies have been shown to significantly reduce OHRQoL by affecting self-esteem and social interactions [2,3]. Accordingly, QoL is a pivotal consideration in prioritizing orthodontic care within the broader healthcare spectrum [8]. Recent research underscores the psychological benefits of orthodontic treatment, such as improved confidence and interpersonal relationships, particularly in adolescents [10]. It has been demonstrated that individuals with malocclusions experience worse OHRQoL than those without malocclusions [4,11]. A meta-analysis by Kragt et al. [10], which combined data from 9,293 young individuals with malocclusions and 10,717 without, found that malocclusions increased the likelihood of impaired OHRQoL by 1.74 times. Furthermore, orthodontic treatment during childhood and adolescence has been shown to result in moderate improvements in the emotional and social dimensions of OHRQoL [11]. However, malocclusions, if untreated, can lead to progressive oral health complications, underscoring the necessity for early intervention [10].

In the past 25 years, orthodontic aligners gained significant popularity due to their ease of wear, comfort, and nearly invisible design [12,13]. Systematic reviews and meta-analyses have indicated substantial advancements in the effectiveness of aligners for aligning and straightening teeth [14,15]. Evidence suggests that aligner wearers often report a more favorable OHRQoL compared to individuals wearing traditional fixed appliances, attributed to their esthetic advantages, improved comfort, better oral hygiene maintenance, and reduced dietary restrictions [12,16,17]. However, despite these benefits, the specific factors influencing OHRQoL among aligner wearers remain barely explored.

The present study aimed to investigate the factors associated with OHRQoL in individuals undergoing orthodontic aligner treatment, providing a nuanced understanding of how sociodemographic and clinical variables shape patient-reported outcomes.

## Materials and methods

### Ethical approval and consent to participate

Ethics approval was obtained from the Ethics Committee of the Federal University of Minas Gerais (No. 39216920.0.0000.5149). Participants were informed that participation was voluntary and that they could decline at any time. Written consent was obtained. Individuals aged 18 or older, as well as guardians of participants younger than 18 years, signed an informed consent form. Participants younger than 18 provided their assent by signing an informed assent form.

### Study design, participants, setting, and eligibility criteria

A cross-sectional study was performed with 34 individuals undergoing treatment with orthodontic aligners in the Postgraduate Program in Orthodontics at the Brazilian Association of Dentistry (ABO-MG) in Belo Horizonte, Brazil. Individuals with reported cognitive disorders or craniofacial anomalies were excluded. Data collection took place between 01st November 2021 and 31st October 2023. The study followed the Strengthening the Reporting of Observational Studies in Epidemiology (STROBE) guidelines [18].

To assess whether our sample size was sufficiently powered to detect statistically significant differences, we referenced the study by Al Nazeh et al. [19]. This study used the OHIP (before and after treatment) to compare the OHRQoL of male and female participants. The mean difference in OHIP total scores between males and females before and after treatment was 7.4, with a pooled standard deviation of 6.9. Considering a male-to-female ratio of 1.08 (24/26) in that study, 80% power, and a Type I error rate of 5% (α=0.05), the required sample size was calculated to be 29 participants (14 males and 15 females, or vice versa).

### OHRQoL

The impact of orthodontic aligners on participants’ OHRQoL was evaluated using the Oral Health Impact Profile (OHIP-14) questionnaire. Printed copies were distributed to participants by the same researcher (G.L.N.S.). The original OHIP consists of 49 items [20]; however, a shorted 14-item version (OHIP-14) was developed in 1997 [21]. This questionnaire assesses seven subscales: functional limitation, physical pain, psychological discomfort, physical disability, psychological disability, social disability, and handicap. Responses are rated on a scale from 0 (never) to 4 (always), with higher scores reflecting a more negative perception of OHRQoL [21]. The OHIP-14 has been validated in other languages, including the Portuguese from Brazil [22].

### Factors associated: sex, age, family income, and malocclusion

Before the start of treatment, independent variables were recorded: participants’ age (years), sex, family income, and malocclusion severity. Family income was evaluated in relation to the Brazilian minimum wage at the time of data collection, with the combined income of all economically active family members used to categorize income into two groups: ≤ 5 minimum wages and > 5 minimum wages. Malocclusion severity was assessed by a calibrated dentist using the Dental Aesthetic Index (DAI) [23]. Data on treatment duration (months) were also collected.

### Pilot study

A pilot study was conducted to test the strategy for data collection. Participants in the pilot study were not eligible for the main study. Following the pilot, changes to the methodology were deemed unnecessary.

### Statistical analysis

The Statistical Package for the Social Science (SPSS) software (IBM SPSS Statistics for Windows, version 25.0, Armonk, NY, USA: IBM Corp.) was used for statistical analysis of the data. A descriptive analysis was initially performed to summarize the dataset. Student’s *t*-test was used to evaluate association between dichotomous independent variables (sex and family income) and OHRQoL. Effect sizes (ES) were calculated using methods described by Cohen [24] and Chinn [25]. For continuous independent variables (age, malocclusion severity, and treatment duration), regression analyses were performed to evaluate their relationship with OHRQoL. Odds ratios (OR) and confidence intervals (CI) were calculated. Poisson regression with robust variance was used to identify factors associated with the impact of orthodontic aligners on participants’ OHRQoL. In this model, sex and family income were analyzed as dichotomous variables, while age, malocclusion severity (DAI), and treatment duration were treated as continuous variables. Beta coefficients (Coef.) and standard errors (SE) were calculated, with statistical significance set at *p* < 0.05.

## Results

Among the 34 participants, 18 (52.9%) were male and 16 (47.1%) were female. The mean age was 33.8 (±8.7) years. No significant differences in OHRQoL were observed between males and females (*p* > 0.05), though the ES for psychological discomfort was moderate (0.43), with males reporting higher (more negative perceptions) mean scores compared to females. Participants from families with an income of < 5 wages had significantly higher scores for social disability and the total score than those from families with an income of ≥ 5 (*p* < 0.05). The ES were moderate for psychological discomfort (0.62), physical disability (0.76), and psychological disability (0.64), while higher ES were found for functional limitation (0.93), social disability (0.98), handicap (0.95), and the total score (0.94). An ES close to 0.20 indicated a small magnitude of association, around 0.50 indicated a moderate magnitude of association, and close to 0.80 indicated a large magnitude of association [24,25]. Overall, individuals from families earning < 5 wages reported more negative perceptions than those from higher-income families (Table 1).

**Table 1:** Associations between dichotomous independent variables and oral health-related quality of life with the calculation of mean differences and effect sizes.

	Functional limitation			Physical pain			Psychological discomfort			Physical disability		
	Mean (SD)	MD (95% CI)	ES	Mean (SD)	MD (95% CI)	ES	Mean (SD)	MD (95% CI)	ES	Mean (SD)	MD (95% CI)	ES
**Sex**												
Male	1.00 (1.08)	0.13 (-0.83-1.08)	0.09	2.72 (1.44)	0.03 (-1.06-1.12)	0.01	3.11 (2.24)	-1.05 (-2.72-0.62)	0.43	0.94 (1.05)	-0.19 (-1.15-0.76)	0.14
Female	1.13 (1.62)			2.75 (1.69)			2.06 (2.54)			0.75 (1.65)		
**Income**												
≥5 wages	0.62 (0.59)	1.15 (-0.01-2.30)	0.93	2.62 (1.65)	0.30 (-0.81-1.42)	0.19	2.05 (2.15)	1.49 (-0.18-1.42)	0.62	0.48 (0.68)	0.98 (-0.18-2.15)	0.76
<5 wages	1.77 (1.87)			2.92 (1.38)			3.54 (2.60)			1.46 (1.89)		
	**Psychological disability**			**Social disability**			**Handicap**			**Total score**		
	**Mean (SD)**	**MD (95% CI)**	**ES**	**Mean (SD)**	**MD (95% CI)**	**ES**	**Mean (SD)**	**MD (95% CI)**	**ES**	**Mean (SD)**	**MD (95% CI)**	**ES**
**Sex**												
Male	2.00 (1.28)	-0.75 (-2.05-0.55)	0.39	0.56 (0.98)	0.13 (-0.66-0.93)	0.11	0.72 (0.95)	0.03 (-1.05-1.11)	0.02	11.06 (6.80)	-1.68 (-7.60-4.24)	0.19
Female	1.25 (2.35)			0.69 (1.30)			0.75 (2.01)			9.38 (10.02)		
**Income**												
≥5 wages	1.19 (1.28)	1.19 (-0.35-2.74)	0.64	0.24 (0.43)	0.99^**^ (0.01-1.96)	0.98	0.24 (0.53)	1.30 (-0.03-2.63)	0.95	7.43 (5.14)	7.42^**^ (0.73-14.10)	0.94
<5 wages	2.38 (2.43)			1.23 (1.58)			1.54 (2.18)			14.85 (10.59)		

**Note:** CI, confidence interval; ES, effect size; MD, mean difference; SD, standard deviation.

** Significant difference (*p* < 0.05).

As shown in Table 2, younger individuals had significantly higher scores (more negative perception) for functional limitation, psychological discomfort, physical disability, psychological disability, social disability, and the total score (*p* < 0.05). Participants with more severe malocclusion also had significantly higher scores for across all OHIP-14 subscales and the total score (*p* < 0.05), though the ES were small.

**Table 2:** Association between continuous independent variables and oral health-related quality of life with the calculation of odds ratios and effect sizes.

	Functional limitation			Physical pain			Psychological discomfort			Physical disability		
	OR	95% CI	ES	OR	95% CI	ES	OR	95% CI	ES	OR	95% CI	ES
**Age**	0.94^**^	0.90-0.98	0.03	0.98	0.96-1.01	0.01	0.95^**^	0.92-0.99	0.02	0.92^**^	0.87-0.97	0.04
**DAI**	1.20^**^	1.10-1.30	0.10	1.07^**^	1.02-1.13	0.03	1.13^**^	1.06-1.22	0.06	1.29^**^	1.16-1.44	0.14
**Time**	1.01	0.92-1.08	0.01	1.01	0.97-1.03	0.01	1.03	0.98-1.07	0.01	1.07^**^	1.01-1.13	0.03
	**Psychological disability**			**Social disability**			**Handicap**			**Total score**		
	**OR**	**95% CI**	**ES**	**OR**	**95% CI**	**ES**	**OR**	**95% CI**	**ES**	**OR**	**95% CI**	**ES**
**Age**	0.95^**^	0.91-0.99	0.02	0.90^**^	0.83-0.97	0.05	0.88	0.84-0.93	0.07	0.95^**^	0.92-0.98	0.02
**DAI**	1.19^**^	1.09-1.29	0.09	1.34^**^	1.19-1.52	0.16	1.32^**^	1.12-1.55	0.15	1.17^**^	1.11-1.23	0.08
**Time**	1.01	0.95-1.06	0.01	1.09^**^	1.02-1.16	0.04	1.06	0.99-1.13	0.03	1.02	0.98-1.06	0.01

**Note:** CI, confidence interval; ES, effect size; OR, odds ratio.

** Statistically significant (*p* < 0.05).

Table 3 displays the results of the Poisson regression, incorporating all potential factors influencing the impact of orthodontic aligner wear on OHRQoL. Individuals with higher DAI scores, indicating more severe malocclusion, exhibited significantly higher scores (indicating a more negative perception) in the following subscales: functional limitation [*p* = 0.002, Coef. (SE) = 0.14 (0.04)], physical pain [*p* = 0.001, Coef. (SE) = 0.08 (0.02)], physical disability [*p* = 0.005, Coef. (SE) = 0.20 (0.07)], psychological disability [*p* = 0.003, Coef. (SE) = 0.16 (0.05)], social disability [*p* = 0.001, Coef. (SE) = 0.18 (0.05)], handicap [*p* = 0.037, Coef. (SE) = 0.16 (0.08)], and the total score [*p* = 0.001, Coef. (SE) = 0.12 (0.03)]. For the handicap subscale [*p* = 0.007, Coef. (SE) = -0.07 (0.02)], younger individuals reported significantly higher scores (indicating a more negative perception).

**Table 3:** Factors associated with the impact of orthodontic aligner wearing on the oral health-related quality of life.

	Functional limitation		Physical pain		Psychological disconfort		Physical disability	
	Coef (SE)	*p* value	Coef (SE)	*p* value	Coef (SE)	*p* value	Coef (SE)	*p* value
**Sex**								
Male	0	0.637	0	0.625	0	0.251	0	0.997
Female	0.15 (0.31)		0.09 (0.19)		-0.31 (0.27)		-0.01 (0.45)	
**Age**	-0.03 (0.02)	0.151	-0.01 (0.01)	0.504	-0.01 (0.01)	0.397	-0.01 (0.03)	0.698
**Income**								
≥5 wages	0	0.112	0	0.231	0	0.384	0	0.335
<5 wages	0.52 (0.33)		-0.18 (0.15)		0.25 (0.29)		0.34 (0.36)	
**DAI**	0.14 (0.04)	0.002^**^	0.08 (0.02)	0.001^**^	0.08 (0.05)	0.105	0.20 (0.07)	0.005^**^
**Time**	-0.02 (0.02)	0.209	-0.01 (0.01)	0.382	0.01 (0.01)	0.693	0.02 (0.02)	0.305
	**Psychological disability**		**Social disability**		**Handicap**		**Total score**	
	**Coef (SE)**	***p* value**	**Coef (SE)**	***p* value**	**Coef (SE)**	***p* value**	**Coef (SE)**	***p* value**
**Sex**								
Male	0	0.183	0	0.121	0	0.610	0	0.747
Female	-0.48 (0.36)		0.72 (0.46)		0.33 (0.66)		-0.06 (0.19)	
**Age**	-0.01 (0.02)	0.605	-0.03 (0.04)	0.357	-0.07 (0.02)	0.007^**^	-0.01 (0.01)	0.177
**Income**								
≥5 wages	0	0.257	0	0.089	0	0.081	0	0.150
<5 wages	0.30 (0.26)		0.66 (0.39)		1.05 (0.60)		0.25 (0.17)	
**DAI**	0.16 (0.05)	0.003^**^	0.18 (0.05)	0.001^**^	0.16 (0.08)	0.037^**^	0.12 (0.03)	0.001^**^
**Time**	-0.02 (0.02)	0.274	0.05 (0.02)	0.067	-0.01 (0.03)	0.930	0.01 (0.01)	0.942

**Note:** Coef, coefficient; DAI, Dental Aesthetic Index; SE, standard error.

** Statistically significant (*p* < 0.05).

## Discussion

Evidence indicates that orthodontic aligners have a more positive impact on OHRQoL compared to conventional fixed orthodontic appliances [26,27]. Aligners are generally associated with reduced pain, discomfort, and functional impairment relative to other orthodontic devices [28]. The growing interest in clear aligners within both clinical practice and academic research reflects their expanding prominence in contemporary orthodontics. As noted by Bruni et al. [29], the increasing number of citations and publications on clear aligner treatment underscores its relevance and the considerable attention it has garnered in recent years. However, research specifically examining the factors influencing OHRQoL among individuals undergoing orthodontic aligner treatment remains scarce [30]. Our findings revealed a moderate association between sex and psychological discomfort, particularly among males. Additionally, family income exhibited moderate to large associations with OHRQoL, as individuals from families earning < 5 wages perceived their OHRQoL more negatively compared to those from higher-income families. Notably, malocclusion severity and patient age were significant factors influencing OHRQoL during aligner treatment, with younger individuals and those with more severe malocclusion reporting more negative perceptions of their QoL. These findings suggest that while aligners offer advantages in terms of comfort and esthetics, malocclusion severity and age play critical roles in shaping the overall patient experience. The methodology used in our study aligns with that of previous research [30], which similarity identified marital status and malocclusion severity as factors influencing OHRQoL in aligner wearers. Consistent with our findings, individuals with more severe malocclusion also reported a more negative perception of their OHRQoL [30].

The present study found that individuals with more severe malocclusion reported a more negative perception of pain during orthodontic treatment with aligners. This could be due to the greater intensity of corrections required for severe malocclusion, which may involve more frequent and significant adjustments, leading to increased discomfort [31]. Furthermore, soft tissue adaptation to newly aligned teeth may be more challenging in such cases [32], contributing to a heightened perception of pain. A recent systematic review found that aligners are associated with less pain than fixed appliances during the early stages of treatment, but the difference in pain intensity between the two modalities was not significant at later stages [17]. Thus, while aligners may offer short-term pain relief, the severity of malocclusion and the adaptation process continue to influence the overall experience of discomfort. The physiological mechanisms underlying the effect of these two treatments on OHRQoL have yet to be defined.

Individuals with more severe malocclusion reported greater functional limitations and physical disabilities, likely due to the complex orthodontic mechanics required to correct more serious cases. Everyday activities such as speaking and chewing may become more challenging [33], further exacerbating the negative impact on psychological well-being and social interactions. The esthetic and functional challenges associated with severe malocclusion can lead to insecurity, reduced psychosocial well-being, and difficulties in social adaptation [34]. These concerns may be further compounded by the extended treatment duration required for complex cases [35], as highlighted by recent qualitative research, which found that many individuals focus on the esthetic improvements they hope to achieve through orthodontic treatment [36].

Younger individuals perceived their OHRQoL more negatively than older patients during orthodontic aligner treatment. This may be attributed to young people’s concerns about how wearing orthodontic devices interferes with daily activities, such as eating and speaking, as well as their responsibility to maintain proper oral hygiene [37]. Prior research has identified that esthetic and functional problems, along with pain, are significant factors negatively affecting OHRQoL in both adolescent and adult groups undergoing orthodontic treatment [38]. Additionally, self-esteem and self-image play pivotal roles during transitional periods like adolescence [39], and peer bullying can exacerbate these negative perceptions [40]. Most participants in the present study were young adults (i.e., mean age 33.8 years), and concerns about dental esthetics when entering the labor market are closely tied to this age group, potentially impacting their QoL. Our findings corroborate prior research involving adolescents; Sauer et al. [41] demonstrated that therapy with aligners during the first year of treatment has a positive effect on psychosocial well-being. However, treating malocclusion can result in physical and functional discomfort, insecurity, and social adaptation challenges, which may affect young individuals’ perception of their QoL [41]. Is has been documented that, in addition to self-driven, appearance-related motivations, parents, health professionals, friends, and peers influence young people’s perception of their need for orthodontic treatment. Achieving an ideal dentofacial appearance is closely linked to both social and personal aspirations [36]. In contrast, older adults typically have a more stable outlook and are less affected by esthetic concerns [42], leading to a potentially less negative perception of OHRQOL during orthodontic treatment with aligners. Future research is warranted to evaluate the applicability of these findings to age-stratified populations and their impact on OHRQoL, especially as aligners continue to gain popularity across all age groups.

The association between lower family income and a more negative perception of OHRQoL was significant in our study. Individuals from underprivileged families may face financial difficulties in meeting the costs associated with orthodontic treatment, which can be prohibitively expensive [43]. In addition, rigid work schedules and transportation costs may complicate regular attendance at appointments, leading to prolonged treatment duration, issues with aligner maintenance, and, in some cases, treatment discontinuation [44].

This study’s cross-sectional design limits its ability to establish causal relationships [45] between the factors associated with wearing orthodontic aligners and their impact on OHRQoL. Additionally, the sample was restricted to individuals undergoing orthodontic treatment in a postgraduate program, which may limit the generalizability of the findings to other settings. Since treatments were performed by postgraduate students, there may have variability in the standard of care provided, despite all participants being treated at the same orthodontic service. Furthermore, cultural and behavioral factors, such as variations in self-motivation, oral hygiene practices, and esthetic concerns, which were not formally examined in this study, may influence the nuances of QoL outcomes and should not be overlooked. For instance, patients with higher esthetic expectations may perceive aligner treatment differently from those with primarily functional goals [46], underscoring the need for a more comprehensive assessment in future research.

Understanding individuals’ perspectives on their health and well-being is essential for orthodontists, as these insights can help tailor treatments to address what matters most to patients [8,9]. This is particularly important when considering how sociodemographic and clinical factors, such as age, income level, and malocclusion severity, influence patients’ daily lives and overall well-being. For example, younger patients may experience greater social challenges due to their heightened sensitivity to peer perception, while financial constraints could affect adherence to aligner wear protocols [46]. These factors can significantly impact patient cooperation, treatment adherence, and the risk of treatment abandonment [46]. Identifying the effects of factors associated with orthodontic treatment, such as differences in patient-reported outcomes between aligner and fixed appliance wears, can help inform decision-makers in improving the quality of orthodontic care services. Future longitudinal studies are needed to explore whether causal relationships exist between orthodontic aligner treatment and OHRQoL. Despite efforts to control for known sociodemographic and clinical confounders, the intricate relationship between orthodontic outcomes and OHRQoL may be influenced by residual confounding factors (e.g., psychological well-being and pre-existing patient expectations).

## Conclusions

In summary, malocclusion severity and patient age influenced the OHRQoL of individuals undergoing orthodontic aligner treatment, with sex and family income also playing important roles.

## References

[1] Sischo L, Broder HL. Oral health-related quality of life: what, why, how, and future implications. J Dent Res. 2011;90(11):1264–70. doi: 10.1177/0022034511399918 21422477 PMC3318061

[2] Haag DG, Peres KG, Balasubramanian M, Brennan DS. Oral Conditions and Health-Related Quality of Life: A Systematic Review. J Dent Res. 2017;96(8):864–74. doi: 10.1177/0022034517709737 28581891

[3] Alrashed M, Alqerban A. The relationship between malocclusion and oral health-related quality of life among adolescents: a systematic literature review and meta-analysis. Eur J Orthod. 2021;43(2):173–83. doi: 10.1093/ejo/cjaa051 33009547

[4] Andiappan M, Gao W, Bernabé E, Kandala N-B, Donaldson AN. Malocclusion, orthodontic treatment, and the Oral Health Impact Profile (OHIP-14): Systematic review and meta-analysis. Angle Orthod. 2015;85(3):493–500. doi: 10.2319/051414-348.1 25157973 PMC8612413

[5] Koistinen S, Olai L, Ståhlnacke K, Fält A, Ehrenberg A. Oral health-related quality of life and associated factors among older people in short-term care. Int J Dent Hyg. 2020;18(2):163–72. doi: 10.1111/idh.12424 31782889 PMC7217038

[6] Van Wilder L, Devleesschauwer B, Clays E, De Buyser S, Van der Heyden J, Charafeddine R, et al. The impact of multimorbidity patterns on health-related quality of life in the general population: results of the Belgian Health Interview Survey. Qual Life Res. 2022;31(2):551–65. doi: 10.1007/s11136-021-02951-w 34424487 PMC8847309

[7] Peres MA, Macpherson LMD, Weyant RJ, Daly B, Venturelli R, Mathur MR, et al. Oral diseases: a global public health challenge. Lancet. 2019;394(10194):249–60. doi: 10.1016/S0140-6736(19)31146-8 31327369

[8] Zhou Y, Wang Y, Wang X, Volière G, Hu R. The impact of orthodontic treatment on the quality of life a systematic review. BMC Oral Health. 2014;14:66. doi: 10.1186/1472-6831-14-66 24913619 PMC4060859

[9] Mandava P, Singaraju GS, Obili S, Nettam V, Vatturu S, Erugu S. Impact of self-esteem on the relationship between orthodontic treatment and the oral health-related quality of life in patients after orthodontic treatment - a systematic review. Med Pharm Rep. 2021;94(2):158–69. doi: 10.15386/mpr-1843 34013186 PMC8118222

[10] Kragt L, Dhamo B, Wolvius EB, Ongkosuwito EM. The impact of malocclusions on oral health-related quality of life in children-a systematic review and meta-analysis. Clin Oral Investig. 2016;20(8):1881–94. doi: 10.1007/s00784-015-1681-3 26635095 PMC5069349

[11] Javidi H, Vettore M, Benson PE. Does orthodontic treatment before the age of 18 years improve oral health-related quality of life? A systematic review and meta-analysis. Am J Orthod Dentofacial Orthop. 2017;151(4):644–655. doi: 10.1016/j.ajodo.2016.12.011 28364887

[12] Kau CH, Soh J, Christou T, Mangal A. Orthodontic Aligners: Current Perspectives for the Modern Orthodontic Office. Medicina (Kaunas). 2023;59(10):1773. doi: 10.3390/medicina59101773 37893491 PMC10608554

[13] Bruni A, Serra FG, Gallo V, Deregibus A, Castroflorio T. The 50 most-cited articles on clear aligner treatment: A bibliometric and visualized analysis. Am J Orthod Dentofacial Orthop. 2021;159(4):e343–62. doi: 10.1016/j.ajodo.2020.11.029 33653640

[14] Zheng M, Liu R, Ni Z, Yu Z. Efficiency, effectiveness and treatment stability of clear aligners: A systematic review and meta-analysis. Orthod Craniofac Res. 2017;20(3):127–33. doi: 10.1111/ocr.12177 28547915

[15] Papageorgiou SN, Koletsi D, Iliadi A, Peltomaki T, Eliades T. Treatment outcome with orthodontic aligners and fixed appliances: a systematic review with meta-analyses. Eur J Orthod. 2020;42(3):331–43. doi: 10.1093/ejo/cjz094 31758191

[16] Kaklamanos EG, Makrygiannakis MA, Athanasiou AE. Oral Health-Related Quality of Life throughout Treatment with Clear Aligners in Comparison to Conventional Metal Fixed Orthodontic Appliances: A Systematic Review. Int J Environ Res Public Health. 2023;20(4):3537. doi: 10.3390/ijerph20043537 36834235 PMC9961780

[17] Li Q, Du Y, Yang K. Comparison of pain intensity and impacts on oral health-related quality of life between orthodontic patients treated with clear aligners and fixed appliances: a systematic review and meta-analysis. BMC Oral Health. 2023;23(1):920. doi: 10.1186/s12903-023-03681-w 38001455 PMC10675971

[18] von Elm E, Altman DG, Egger M, Pocock SJ, Gøtzsche PC, Vandenbroucke JP, et al. Strengthening the Reporting of Observational Studies in Epidemiology (STROBE) statement: guidelines for reporting observational studies. BMJ. 2007;335(7624):806–8. doi: 10.1136/bmj.39335.541782.AD 17947786 PMC2034723

[19] Al Nazeh AA, Alshahrani I, Badran SA, Almoammar S, Alshahrani A, Almomani BA, et al. Relationship between oral health impacts and personality profiles among orthodontic patients treated with Invisalign clear aligners. Sci Rep. 2020;10(1):20459. doi: 10.1038/s41598-020-77470-8 33235288 PMC7686374

[20] Slade GD, Spencer AJ. Development and evaluation of the Oral Health Impact Profile. Community Dent Health. 1994;11(1):3–11. 8193981

[21] Slade GD. Derivation and validation of a short-form oral health impact profile. Community Dent Oral Epidemiol. 1997;25(4):284–90. doi: 10.1111/j.1600-0528.1997.tb00941.x 9332805

[22] Oliveira BH, Nadanovsky P. Psychometric properties of the Brazilian version of the Oral Health Impact Profile-short form. Community Dent Oral Epidemiol. 2005;33(4):307–14. doi: 10.1111/j.1600-0528.2005.00225.x 16008638

[23] Jenny J, Cons NC. Establishing malocclusion severity levels on the Dental Aesthetic Index (DAI) scale. Aust Dent J. 1996;41(1):43–6. doi: 10.1111/j.1834-7819.1996.tb05654.x 8639114

[24] Cohen J. Statistical Power Analysis for the Behavioral Sciences. 2nd ed. Hillsdale, NJ: Lawrence Erlbaum Associates; 1988.

[25] Chinn S. A simple method for converting an odds ratio to effect size for use in meta-analysis. Statist Med. 2000;19(22):3127–31. doi: 10.1002/1097-0258(20001130)19:22<3127::aid-sim784>3.0.co;2-m 11113947

[26] Alfawal AMH, Burhan AS, Mahmoud G, Ajaj MA, Nawaya FR, Hanafi I. The impact of non-extraction orthodontic treatment on oral health-related quality of life: clear aligners versus fixed appliances-a randomized controlled trial. Eur J Orthod. 2022;44(6):595–602. doi: 10.1093/ejo/cjac012 35395075

[27] AlSeraidi M, Hansa I, Dhaval F, Ferguson DJ, Vaid NR. The effect of vestibular, lingual, and aligner appliances on the quality of life of adult patients during the initial stages of orthodontic treatment. Prog Orthod. 2021;22(1):3. doi: 10.1186/s40510-020-00346-0 33458787 PMC7811964

[28] Cardoso PC, Espinosa DG, Mecenas P, Flores-Mir C, Normando D. Pain level between clear aligners and fixed appliances: a systematic review. Prog Orthod. 2020;21(1):3. doi: 10.1186/s40510-019-0303-z 31956934 PMC6970090

[29] Bruni A, Serra FG, Gallo V, Deregibus A, Castroflorio T. The 50 most-cited articles on clear aligner treatment: A bibliometric and visualized analysis. Am J Orthod Dentofacial Orthop. 2021;159(4):e343–62. doi: 10.1016/j.ajodo.2020.11.029 33653640

[30] Zamora-Martínez N, Paredes-Gallardo V, García-Sanz V, Gandía-Franco JL, Tarazona-Álvarez B. Comparative Study of Oral Health-Related Quality of Life (OHRQL) between Different Types of Orthodontic Treatment. Medicina (Kaunas). 2021;57(7):683. doi: 10.3390/medicina57070683 34356964 PMC8304849

[31] Chow J, Cioffi I. Pain and orthodontic patient compliance: A clinical perspective. Seminars in Orthodontics. 2018;24(2):242–7. doi: 10.1053/j.sodo.2018.04.006

[32] Maetevorakul S, Viteporn S. Factors influencing soft tissue profile changes following orthodontic treatment in patients with Class II Division 1 malocclusion. Prog Orthod. 2016;17:13. doi: 10.1186/s40510-016-0125-1 27135067 PMC4852168

[33] Babaee Hemmati Y, Mirmoayed A, Ghaffari ME, Falahchai M. Eating- and oral health-related quality of life in patients under fixed orthodontic treatment. Clin Exp Dent Res. 2022;8(5):1192–201. doi: 10.1002/cre2.631 35809225 PMC9562793

[34] Dimberg L, Arnrup K, Bondemark L. The impact of malocclusion on the quality of life among children and adolescents: a systematic review of quantitative studies. Eur J Orthod. 2015;37(3):238–47. doi: 10.1093/ejo/cju046 25214504

[35] Mavreas D, Athanasiou AE. Factors affecting the duration of orthodontic treatment: a systematic review. Eur J Orthod. 2008;30(4):386–95. doi: 10.1093/ejo/cjn018 18678758

[36] Prado LH, Previato K, Delgado RZR, Nelson Filho P, Bezerra Segato RA, Nakane Matsumoto MA, et al. Adolescents’ perception of malocclusion, their motivations, and expectations concerning the orthodontic treatment. Is it all about attractiveness? A qualitative study. Am J Orthod Dentofacial Orthop. 2022;161(4):e345–52. doi: 10.1016/j.ajodo.2021.10.014 35031194

[37] Corradi-Dias L, Paiva SM, Pretti H, Pordeus IA, Abreu LG. Impact of the onset of fixed appliance therapy on adolescents’ quality of life using a specific condition questionnaire: A cross-sectional comparison between male and female individuals. J Orthod. 2019;46(3):195–204. doi: 10.1177/1465312519851220 31144564

[38] Paes da Silva S, Pitchika V, Baumert U, Wehrbein H, Schwestka-Polly R, Drescher D, et al. Oral health-related quality of life in orthodontics: a cross-sectional multicentre study on patients in orthodontic treatment. Eur J Orthod. 2020;42(3):270–80. doi: 10.1093/ejo/cjz064 31605613

[39] Sawyer SM, Azzopardi PS, Wickremarathne D, Patton GC. The age of adolescence. Lancet Child Adolesc Health. 2018;2(3):223–8. doi: 10.1016/S2352-4642(18)30022-1 30169257

[40] Broutin A, Blanchet I, Canceill T, Noirrit-Esclassan E. Association between Dentofacial Features and Bullying from Childhood to Adulthood: A Systematic Review. Children (Basel). 2023;10(6):934. doi: 10.3390/children10060934 37371166 PMC10297497

[41] Sauer MK, Drechsler T, Peron PF, Schmidtmann I, Ohlendorf D, Wehrbein H, et al. Aligner therapy in adolescents: first-year results on the impact of therapy on oral health-related quality of life and oral hygiene. Clin Oral Investig. 2023;27(1):369–75. doi: 10.1007/s00784-022-04741-1 36308561 PMC9876846

[42] Kavand G, Broffitt B, Levy SM, Warren JJ. Comparison of dental esthetic perceptions of young adolescents and their parents. J Public Health Dent. 2012;72(2):164–71. doi: 10.1111/j.1752-7325.2011.00306.x 22364682 PMC3370263

[43] Ghonmode S, Shrivastava S, Kadaskar AR, Bapat S. Socioeconomic burden of orthodontic treatment: a systematic review. Med Pharm Rep. 2023;96(2):154–63. doi: 10.15386/mpr-2457 37197274 PMC10184526

[44] Price J, Whittaker W, Birch S, Brocklehurst P, Tickle M. Socioeconomic disparities in orthodontic treatment outcomes and expenditure on orthodontics in England’s state-funded National Health Service: a retrospective observational study. BMC Oral Health. 2017;17(1):123. doi: 10.1186/s12903-017-0414-1 28927396 PMC5605975

[45] Wang X, Cheng Z. Cross-Sectional Studies: Strengths, Weaknesses, and Recommendations. Chest. 2020;158(1S):S65–71. doi: 10.1016/j.chest.2020.03.012 32658654

[46] Marañón-Vásquez GA, Barreto LS da C, Pithon MM, Nojima LI, Nojima M da CG, Araújo MT de S, et al. Reasons influencing the preferences of prospective patients and orthodontists for different orthodontic appliances. Korean J Orthod. 2021;51(2):115–25. doi: 10.4041/kjod.2021.51.2.115 33678627 PMC7940807

